# TRIpartite Motif 21 (TRIM21) Differentially Regulates the Stability of Interferon Regulatory Factor 5 (IRF5) Isoforms

**DOI:** 10.1371/journal.pone.0103609

**Published:** 2014-08-01

**Authors:** Elisa Lazzari, Justyna Korczeniewska, Joan Ní Gabhann, Siobhán Smith, Betsy J. Barnes, Caroline A. Jefferies

**Affiliations:** 1 Molecular and Cellular Therapeutics, Research Institute, Royal College of Surgeons in Ireland, Dublin, Ireland; 2 Department of Biochemistry and Molecular Biology, Rutgers Biomedical and Health Sciences, Newark, New Jersey, United States of America, Rutgers Biomedical and Health Sciences, New Jersey Medical School- Cancer Center, Newark, New Jersey, United States of America; University of Nebraska-Lincoln, United States of America

## Abstract

IRF5 is a member of the Interferon Regulatory Factor (IRF) family of transcription factors activated downstream of the Toll-Like receptors (TLRs). Polymorphisms in *IRF5* have been shown to be associated with the autoimmune disease Systemic Lupus Erythematosus (SLE) and other autoimmune conditions, suggesting a central role for IRF5 in the regulation of the immune response. Four different IRF5 isoforms originate due to alternative splicing and to the presence or absence of a 30 nucleotide insertion in *IRF5* exon 6. Since the polymorphic region disturbs a PEST domain, a region associated with protein degradation, we hypothesized that the isoforms bearing the insertion might have increased stability, thus explaining the association of individual IRF5 isoforms with SLE. As the E3 ubiquitin ligase TRIpartite Motif 21 (TRIM21) has been shown to regulate the stability and hence activity of members of the IRF family, we investigated whether IRF5 is subjected to regulation by TRIM21 and whether dysregulation of this mechanism could explain the association of IRF5 with SLE. Our results show that IRF5 is degraded following TLR7 activation and that TRIM21 is involved in this process. Comparison of the individual IRF5 variants demonstrates that isoforms generated by alternative splicing are resistant to TRIM21-mediated degradation following TLR7 stimulation, thus providing a functional link between isoforms expression and stability/activity which contributes to explain the association of IRF5 with SLE.

## Introduction

Systemic lupus erythematosus (SLE) is a chronic autoimmune disease characterised by a complex interplay between innate and adaptive immune systems. Nucleic acid sensing receptors such as TLR7 and TLR9, which recognise RNA and DNA, respectively, have been shown to contribute to autoantibody and type I interferon (IFN) production in SLE [1]–[4]. In this context the transcription factor IRF5, which promotes pro-inflammatory cytokines and type I IFN production in response to both TLR7 and -9 activation, has been genetically and functionally associated with SLE [5]–[7].

Polymorphisms in the *IRF5* gene define haplotypes that can have a protective or exacerbating (risk) effect on lupus susceptibility, with the risk haplotype being characterized by the presence of Single Nucleotide Polymorphisms (SNPs) in the promoter region and 3′ untranslated region (UTR) which result in enhanced levels of IRF5 mRNA. In addition, different isoforms of the IRF5 protein are expressed due to the presence or absence of a 30 nucleotide insertion in exon 6, which has also been included in the risk haplotype. Furthermore, *IRF5* exon 6 contains an alternative splice site 48 nucleotides downstream of the 5′ end, and different combinations of insertion/deletion and alternative/conventional splicing lead to the expression of four IRF5 isoforms (IRF5-V1, -V2, -V3 and -V5) presenting different deletion patterns in their central region. Since *IRF5* exon 6 encodes for part of a PEST domain normally present in proteins characterised by rapid turnover, one hypothesis is that the presence or absence of the insertion and the mechanism of splicing may influence the stability of the different IRF5 isoforms [8]–[11]. Indeed, enhanced levels of IRF5 mRNA and proteins were observed in Peripheral Blood Mononuclear Cells (PBMCs) from SLE patients and importantly, the increased levels of IRF5 correlates with elevated levels of circulating IFNα, thus highlighting the link between *IRF5* genotype and dysregulation of IRF5 function and consequentially of type I IFN expression [9], [10], [12]–[15].

The E3 ubiquitin ligase TRIM21 plays an important role in regulating the stability and hence activity of the IRF family of transcription factors. TRIM21 is in fact able to interact with IRF3, IRF7 and IRF8 upon TLR stimulation, resulting in TRIM21-mediated ubiquitination, subsequent degradation and hence termination of signaling [16]–[18]. Although the role of TRIM21 as a negative regulator of IRF-mediated responses is well established, recent studies demonstrate that in resting cells and in the early phase of the immune response TRIM21 may act to enhance IRF3 and IRF8 transcriptional activity, while other factors may cooperate with TRIM21 for IRF degradation in the late phase of signaling [17], [19], [20]. Regardless of the specific molecular mechanisms involved, the importance of TRIM21 as a regulator if IFN responses is nonetheless demonstrated by the severe “lupus-like” disease developed by *Trim21* knock out mice, characterized by enhanced production of pro-inflammatory cytokines such as type I interferons, IL-12 and IL-23, all of which are known to be regulated by IRF family members [16], [18], [20]–[22].

In this context, investigating the interplay between IRF5 and TRIM21 and the stability of individual IRF5 isoforms is of particular relevance for understanding the *IRF5* risk haplotype and the contribution of IRF5 to the disease. Given that exon 6 encodes for a Proline-, Glutamic acid-, Serine-, Threonine-rich (PEST) domain potentially important for IRF5 stability, we hypothesized that the various IRF5 isoforms generated from insertion/deletion and/or alternative splicing may have altered stability, potentially as a result of altered ability to interact with TRIM21, and hence downstream effects on IRF5-mediated gene transcription. We demonstrated that IRF5 can directly interact with TRIM21 and interestingly the interaction is inducible upon TLR7 stimulation, thus suggesting that TRIM21 may target IRF5 in this pathway. Contrary to our initial hypothesis, mapping of IRF5 domains involved in the interaction revealed that the IRF5 polymorphic region is dispensable for the association between IRF5 and TRIM21, and in fact we demonstrate that all of the IRF5 isoforms investigated in this study interact with TRIM21 to a similar extent. In determining the functional consequences of this interaction we observed that IRF5 isoforms originating from alternative splicing (IRF5-V2 and IRF5-V3) are resistant to TRIM21-mediated degradation whereas IRF5-V1 and IRF5-V5 are targeted for TRIM21-mediated degradation in TLR7-stimulated cells. The inability of TRIM21 to degrade IRF5-V2 and IRF5-V3 results in abrogation of TRIM21-mediated inhibition of IRF5-driven reporter activity, and corresponds with previously reported enhanced expression and activity in SLE [23]. Altogether, these results demonstrate that dysregulation of the IRF5-TRIM21 regulatory loop or expression of more stable isoforms in SLE patients could represent a novel mechanism of pathogenesis in SLE and possibly other autoimmune diseases.

## Materials and Methods

### Cell culture

Human embryonic kidney (HEK)-293T (ECACC, United Kingdom) and HEK-TLR7 (InvivoGen) cells were cultured in Dulbecco’s Modified Essential Medium (DMEM) supplemented with 10% (v/v) heat inactivated fetal calf serum (FCS), 100 units/ml Penicillin and 100 µg/ml Streptomycin. Blasticidin (InvivoGen) was added to a final concentration of 10 µg/ml for culture of HEK-TLR7. THP-1 cells (ECACC, United Kingdom) were cultured in RPMI-1640 supplemented with 10% (v/v) fetal calf serum and 100 units/ml Penicillin and 100 µg/ml Streptomycin. All cells were maintained at 37°C in 5% CO_2_. Primary human peripheral blood mononuclear cells (PBMCs) were isolated from whole blood from healthy donors, under ethical approval from Royal College of Surgeons in Ireland research ethics committee REC269, using a Ficoll gradient and cultured in RPMI-1640 media supplemented with 10% (v/v) heat inactivated fetal calf serum and 100 units/ml Penicillin and 100 µg/ml Streptomycin. Informed consent from all participants involved in this study was obtained in a written manner. Participants involved in this study were only recruited from, and experimentation conducted at, Royal College of Surgeons in Ireland.

### Plasmids and reagents

Plasmids encoding Myc-tagged IRF5 isoforms were a kind gift of Dr. Frank Neipel (Virologisches Institut - Klinische und Molekulare Virologie, Erlangen, Germany). Plasmids encoding Xpress-TRIM21 and GST-TRIM21 PRY/SPRY domain were a gift from Dr. David Rhodes (Cambridge Institute for Medical Research, Cambridge, UK). HA-ubiquitin wild type and mutants were a gift from Dr. James Burrows (Centre for Cancer Research and Cell Biology, Belfast, UK). Plasmids encoding FLAG-tagged IRF5 full length and deletion mutants were described previously [24]. Myc-MyD88 construct was a kind gift from Dr. Alberto Mantovani (Istituto Clinico Humanitas, Milan, Italy). pGL3-IFNA4 luciferase and pGL4-TK-Renilla were a kind gift from Dr. John Hiscott (Lady Davis Institute, Montreal, Canada) and Dr. Kate Fitzgerald (UMASS, Massachusetts, USA), respectively. Plasmids encoding shRNA targeting TRIM21 and scrambled negative control were described previously [16]. TLR ligands were purchased from InvivoGen (California, USA). Primary antibodies used were anti-FLAG (Sigma), anti-c-Myc and anti-β-Actin (Abcam), anti-GST (GE Healthcare), anti-Xpress (Invitrogen), anti-IRF5 (Cell Signaling) and anti-α-actinin, anti-HA and anti-TRIM21 (Santa Cruz).

### Immunoprecipitation and western blot analysis

Immunoblots were performed as previously described [16]. For immunoprecipitations, cells were transfected as indicated and lysed in RIPA buffer (PBS containing 0.5% (w/v) sodium deoxycholate, 0.1% (w/v) SDS and 1% (v/v) Nonidet P40) supplemented with protease inhibitors (PMSF 1 mM, Na_3_VO_4_ 1 mM, KF 1 mM, Pepstatin A 1 µg/ml and Leupeptin HCl 1 µg/ml). Cleared cell lysates were incubated with HA-agarose (Sigma) or with 1 µg of anti-Xpress antibody followed by incubation with protein G sepharose (GE Healthcare). For recombinant pulldowns, cells were lysed in Tris-HCl lysis buffer (50 mM Tris-HCl pH 7.4, 1% (v/v) Nonidet P40, 0.25% (w/v) sodium deoxycholate, 150 mM NaCl, 1 mM EDTA) supplemented with protease inhibitors and incubated with 1 µg of Glutathione S-Transferase (GST) or GST-PRY/SPRY TRIM21 bound to glutathione agarose (Qiagen). Isolated proteins were separated by 10% SDS-PAGE.

### Real-time polymerase chain reaction (PCR)

RNA was extracted from cell cultures using TRIzol reagent (Sigma) and reverse transcribed to complementary DNA using Tetro cDNA synthesis kit (Bioline) according to the manufacturer’s recommendations. Real-time quantitative PCR was performed with SYBR Green Taq ReadyMix (Sigma), using the following primer pairs for human IL-6: sense 5′-AGTTCCTGCAGAAAAAGGCA-3′ and antisense 5′-AAAGCTGCGCAGAATGAGAT-3′ and human 18sRNA: sense 5′-GGGAGGTAGTGACGAAAAAT-3′ and antisense 5′-ACCAACAAAATAGAACCGCG-3′. Data were analyzed using an ABI Prism 7900 system (Applied Biosystems) and were normalized to a GAPDH reference. Real-time PCR data were analyzed using the 2^−ΔΔCt^ method [25].

### Pulse-chase experiments to determine IRF5 stability

HEK-TLR7 cells transfected with individual isoforms were pre-treated for 30 minutes with cycloheximide (100 µg/ml, Sigma) followed by stimulation with CL097 (5 µg/ml). Samples were harvested after 4 and 8 hours of treatment and proteins resolved by 10% SDS-PAGE. Densitometric analysis was performed using GeneTools software (Syngene) in order to calculate the ratio between IRF5 and α-actinin levels in each sample.

### Confocal microscopy

HeLa cells were transfected with 500 ng of GFP-IRF5 and 500 ng of mRFP-TRIM21 for 18–24 hr and were then treated with Imiquimod (20 µg/ml) for 3 hr. Cells were fixed with 4% paraformaldehyde (Sigma) and mounted in DAPI containing mounting media (Dako). Cells were imaged by confocal microscopy on a Zeiss LSM 510 META (Oberkochen, Germany).

### Reporter gene assay

HEK-293T (1×10^4^ per well) were seeded in a 96-well plate 24 hours prior to transfection with 50 ng of reporter gene (firefly luciferase controlled by the IFNA4 promoter) and 50 ng IRF5 in presence of increasing amounts (10–100 ng) of Xpress-TRIM21, or 50 ng MyD88 and 100 ng Xpress-TRIM21. Renilla luciferase (5 ng) was used as internal control. All transfections were carried out using Metafectene (Biontex) according to the manufacturer’s instructions. Luciferase activity was analyzed 48 hours post-transfection and standardized to Renilla luciferase activity to normalize for transfection efficiency.

## Results

### TRIM21 interacts with IRF5 and regulates its stability and activity

**Figure 1:**
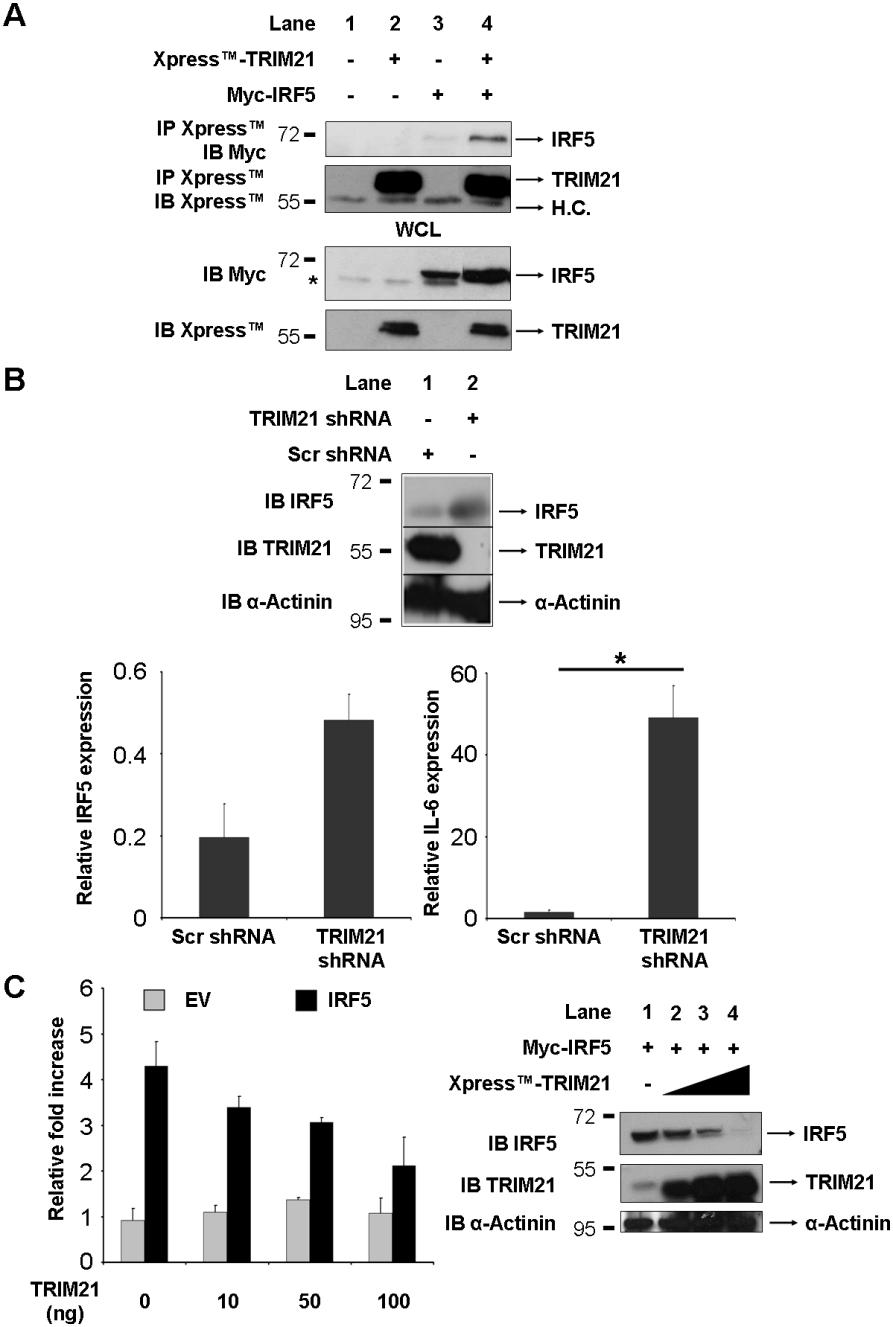
TRIM21 interacts with IRF5 and regulates its stability and activity. A, Myc-IRF5 and Xpress-TRIM21 were overexpressed in HEK-293T cells. 24 hours post-transfection cells were lysed, Xpress-TRIM21 was immunoprecipitated from cell lysates and association of TRIM21 with IRF5 was assessed by anti-Myc immunoblot. WCL, whole cell lysate; H.C., Heavy Chain; *indicates non-specific signal. B, HEK-293T cells were transfected with 2 µg of plasmids encoding shRNA targeting TRIM21 or scrambled shRNA as a negative control. 48 hours after transfection cells were lysed and levels of IRF5, TRIM21 and α-actinin were determined by western blot. Bottom graphs show densitometric analysis of relative IRF5 levels (left) and expression of IL-6 as determined by RT-PCR of RNA extracted from the same samples (right). C, HEK293T were transfected with plasmids encoding the luciferase reporter gene under the control of the IFNA4 promoter and Myc-tagged IRF5 in presence of increasing amounts of Xpress-TRIM21. The TK-Renilla plasmid was used as internal control. Luciferase activity was measured 48 hours after transfection and normalized to renilla activity. Results are shown as fold activation over Empty Vector control. Expression of IRF5, TRIM21 and α-actinin was determined by western blot with anti-Myc, anti-Xpress and anti-α-actinin, respectively. *p<0.05 as determined by Student’s *t*-test.

Regulation of transcription factor turnover is an important mechanism to control gene expression. The E3 ubiquitin ligase TRIM21 plays a major role in regulating the immune response by controlling stability and activity of various members of the interferon regulatory factor family. Interestingly, the ability of TRIM21 to ubiquitinate IRF5 has previously been demonstrated, but the effects of this post-translational modification on IRF5 stability and activity have yet to be elucidated [18]. We first investigated whether IRF5 and TRIM21 could interact directly *in vivo* by overexpressing plasmids encoding Myc-tagged IRF5 and Xpress-tagged TRIM21 in HEK-293T cells followed by immunoprecipitation of TRIM21 from cell lysates. As figure 1A shows, blotting of immunocomplexes with anti-Myc revealed a direct interaction between the two proteins (figure 1A, upper panel, lane 4).

We next assessed the effect of TRIM21 depletion on IRF5 stability and activity. HEK-293T cells were transfected with shRNA targeting TRIM21 or scrambled shRNA as control for off-target effects. Western blot analysis of IRF5 levels shows a marked increase in IRF5 expression in cells depleted of TRIM21, thus indicating that TRIM21 has a negative effect on IRF5 stability (figure 1B, upper panel, compare lane 2 with lane 1), as confirmed by densitometric analysis (figure 1B, bottom panel, left). Accordingly, the expression of an IRF5-controlled gene, IL-6 [6], was found to be dramatically enhanced in absence of TRIM21, in keeping with the elevated levels of IRF5 observed in these samples (figure 1B, bottom panel, right). To confirm that TRIM21 can negatively regulate IRF5 transcriptional activity, we examined the effect of TRIM21 on the ability of one of the IRF5 isoforms examined in this study, IRF5-V1 (described below), to activate an IFNA4-dependent promoter. As figure 1C shows, the activity of IRF5 is dose-dependently inhibited by TRIM21 (figure 1C, left), and the reduction in activity results from TRIM21-mediated degradation of IRF5 as shown by western blot performed on lysates from the corresponding samples (figure 1C, right).

### The IRF association domain of IRF5 interacts with TRIM21 via its PRY/SPRY domain

**Figure 2:**
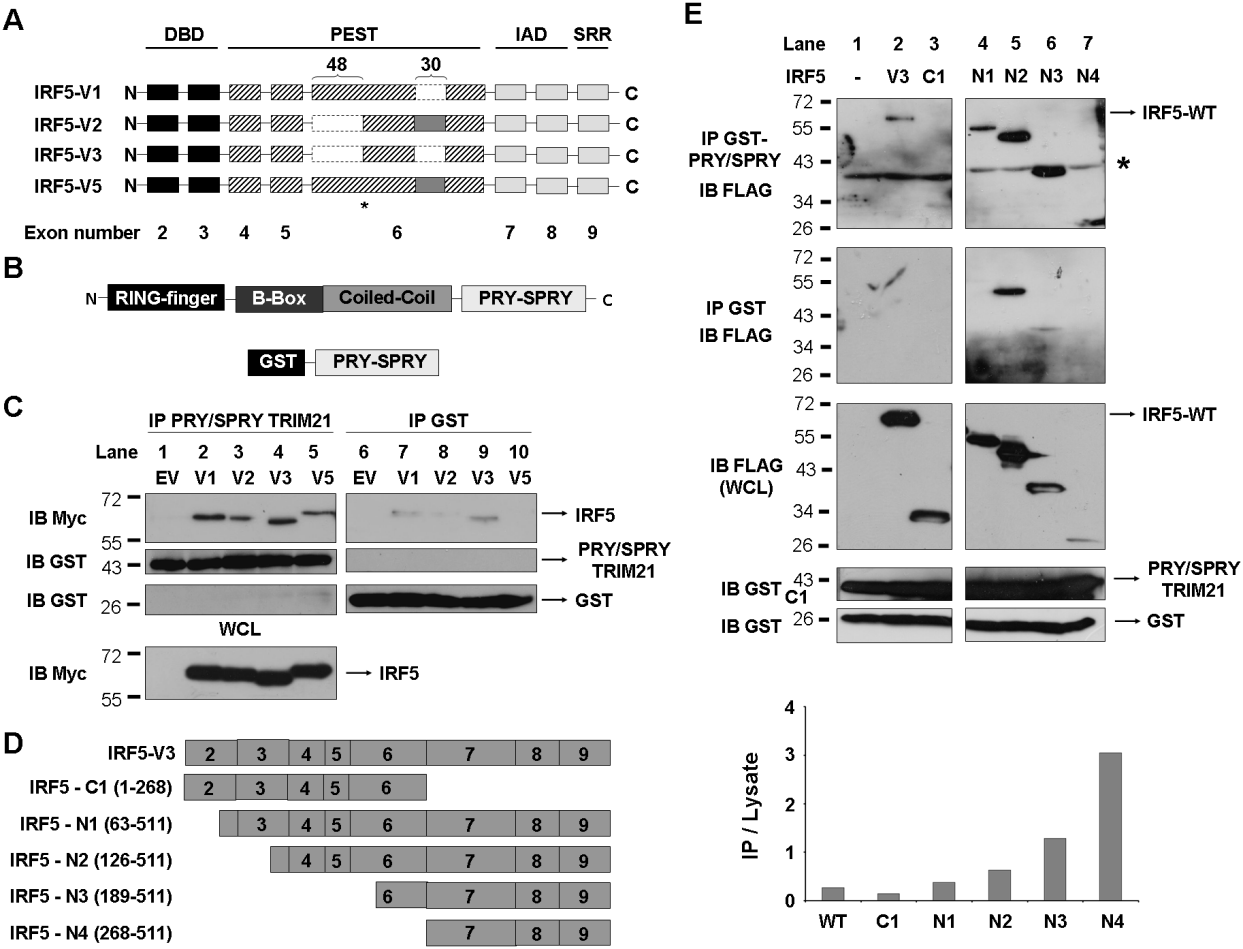
Analysis of interaction domains of IRF5 and TRIM21. A, Exon schematic of IRF5 isoforms structure. DBD, DNA binding domain; PEST, region rich in proline (P), glutamic acid (E), serine (S) and threonine (T) residues; IAD, IRF association domain; SRR, Serine-Rich Region. Dotted lines represent deleted regions. The dark grey box in exon 6 represents the polymorphic 30 nucleotide insertion while *indicates the position of the alternative splicing site 48 nucleotides from exon 6 5′ end. B, Domain structure of TRIM21 (top) and GST-tagged PRY/SPRY domain (bottom). C, Myc-IRF5 isoforms were overexpressed in HEK-293T and lysates were incubated with GST-PRY/SPRY TRIM21 (left panel) or GST alone (right panel) bound to glutathione agarose. Interaction of IRF5 isoforms was assessed by immunoblot (top panels) and total IRF5 expression in the whole cell lysate (WCL) is shown in the bottom panel. D, Schematic diagram of exons encoding full length IRF5-V3 (top) and exons deletions originating C-terminal (C1) or N-terminal (N1–N4) truncated proteins. E, Full length FLAG-IRF5 or deletion mutants were overexpressed in HEK-293T and lysates were incubated with GST-PRY/SPRY TRIM21 (top panel) or GST alone bound to glutathione agarose. Interaction of IRF5 was assessed by anti-FLAG immunoblot and total IRF5 expression in the whole cell lysate (WCL) is shown. Anti-GST immunoblots (bottom panels) show amount of GST-PRY/SPRY TRIM21 or GST incubated with cell lysates. *indicates non-specific signal. Band intensity was calculated and ratio between pulled-down signal and total expression in the whole cell lysate is shown (bottom graph).

Having shown that TRIM21 can interact with IRF5 and has an effect on its stability and activity, we next sought to investigate which domains in IRF5 and TRIM21 were important to mediate this interaction. We first assessed the ability of IRF5 variants arising from the combination of insertion/deletion and alternative use of the 5′ splice site in exon 6 (shown in figure 2A and hereafter referred to as IRF5-V1, -V2, -V3 and -V5) to interact *in vitro* with recombinant GST-tagged TRIM21 PRY/SPRY domain (figure 2B), previously shown to be necessary for interaction with its identified substrates such as IRF3, IRF8 and DDX41 [16], [17], [19], [26], [27]. Lysates from HEK-293T cells overexpressing Myc-tagged IRF5 isoforms were incubated with GST-tagged TRIM21 PRY/SPRY domain or GST alone as a negative control and proteins were resolved by SDS-PAGE. Figure 2C (upper panel, lanes 2–5) shows that all the isoforms interact with TRIM21 PRY/SPRY domain in a similar manner, suggesting that polymorphisms in the region encoded by exon 6 in IRF5 do not affect IRF5-TRIM21 interaction and confirming that the C-terminal PRY/SPRY domain in TRIM21 can mediate the interaction between the two proteins.

In order to determine which domain in IRF5 was necessary for the interaction and to further investigate the possible involvement of IRF5 polymorphic region in mediating the association with TRIM21, we next assessed the interaction properties of TRIM21 with full length IRF5 or various IRF5 deletion mutants (as outlined in figure 2D). Like other IRF family members, IRF5 is composed of a conserved N-terminal DNA binding domain, a central linker region/PEST domain and a C-terminal IRF Association Domain (IAD) known to mediate interaction of IRF5 with transcriptional activators such as CBP/p300 [28]. We therefore incubated lysates from HEK-293T overexpressing full length or truncated variants of IRF5 with recombinant GST-tagged TRIM21 PRY/SPRY domain or GST alone as a negative control. As figure 2E shows, interaction between recombinant TRIM21 PRY/SPRY and an IRF5 mutant lacking the C-terminal IAD (IRF5-C1) was nearly completely abolished as compared to the full length IRF5 protein, thus demonstrating that the IAD domain is critically important for mediating protein-protein interactions in IRF5 (figure 2E, upper panel, lane 3). Indeed, we observed that the IAD domain of IRF5 alone, encoded by exons 7 through 9, could interact with recombinant TRIM21 PRY/SPRY (figure 2E, upper panel, lane 7), thus indicating that the IAD is sufficient to mediate IRF5-TRIM21 interaction. Interestingly we observed that, as compared to IRF5 full length, mutants bearing N-terminal truncations (IRF5-N1-N3) showed enhanced interaction with TRIM21, thus suggesting that IRF5 N-terminal domains may have an inhibitory effect on this interaction (figure 2E, upper panel, lanes 4–6 and corresponding densitometry graph, bottom panel). Taken together, these results indicate that an intact C-terminal IAD domain of IRF5 is required and sufficient to mediate the interaction with the PRY/SPRY domain of TRIM21 (figure 2E, upper panel, lane 7), indicating therefore that the polymorphic region encoded by exon 6 is not directly involved in binding TRIM21. Furthermore, these experiments confirm that the C-terminal PRY/SPRY domain in TRIM21 can mediate the association with IRF5, in keeping with an increasing body of evidence indicating that the C-terminal region represents the substrate interaction domain in TRIM21 [29].

### Different IRF5 isoforms interact with TRIM21 equally upon TLR7 stimulation and act as substrates for TRIM21-mediated ubiquitination

**Figure 3:**
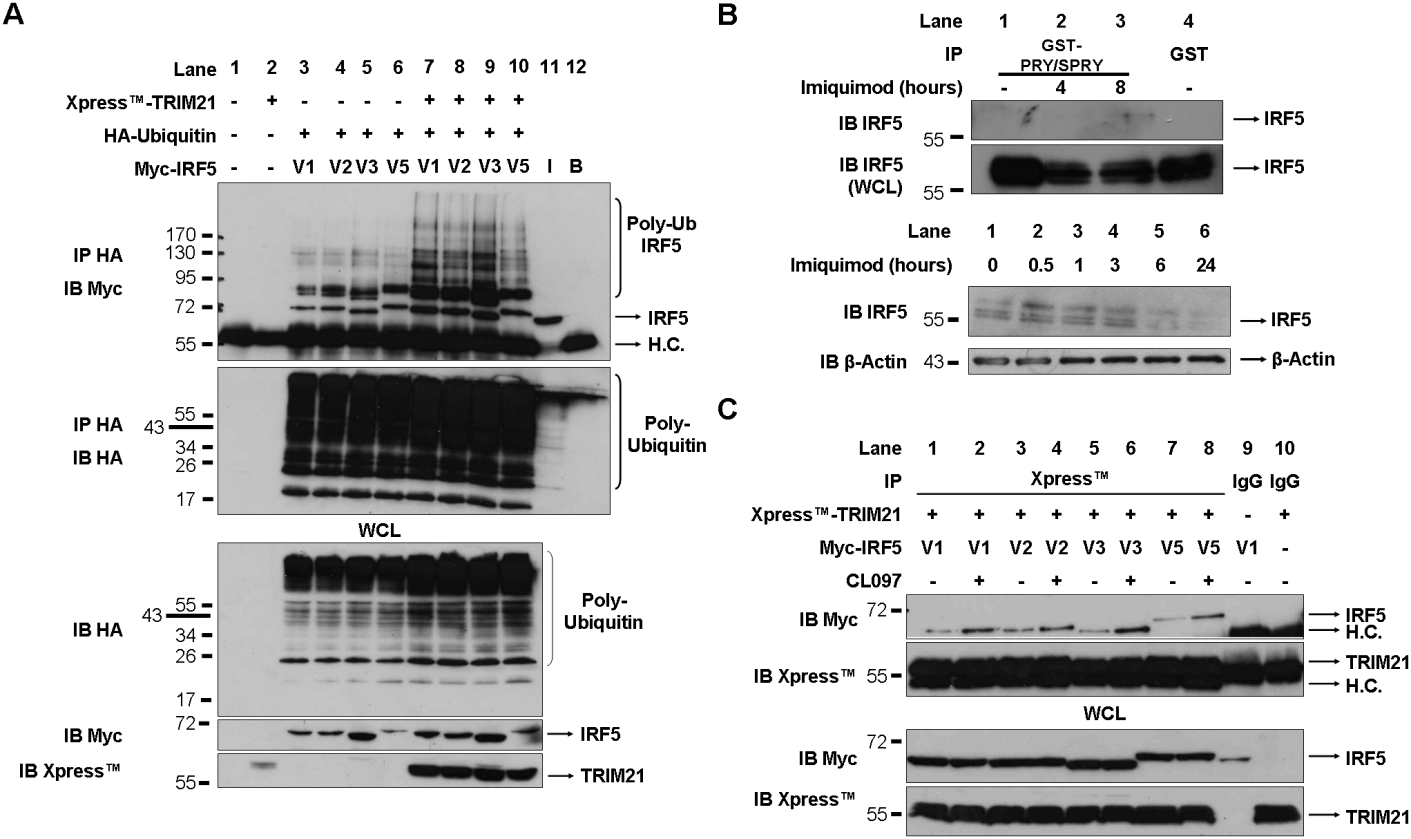
TRIM21 ubiquitinates IRF5 and interacts with IRF5 isoforms upon TLR7 stimulation. A, Myc-tagged IRF5 isoforms and HA-Ubiquitin were overexpressed in HEK-293T in presence or absence of Xpress-TRIM21. Lysates were incubated with HA agarose and the extent of IRF5 ubiquitination was assessed by anti-Myc immunoblot (top panel). Expression of IRF5 and TRIM21 in the Whole Cell Lysate (WCL) is shown in the bottom panels. I (lane 11), Myc-IRF5-V1 Input; B (lane 12), HA-agarose beads alone; H.C., Heavy Chain. B, Top panel: THP-1 were stimulated with Imiquimod (10 µg/ml) for 4 and 8 hours and lysates were incubated with GST-PRY/SPRY TRIM21 (lanes 1–3) or GST alone (lane 4) bound to glutathione agarose. Interaction of IRF5 and total IRF5 expression in the whole cell lysate (WCL) was assessed by immunoblot. Bottom panel: PBMCs were treated with 10 µg/ml Imiquimod for the indicated times. Proteins were resolved by SDS PAGE and immunoblotting performed with anti-IRF5 and anti-β-Actin antibodies. C, Myc-IRF5 isoforms and Xpress-TRIM21 were overexpressed in HEK-TLR7 cells. Following 8 hours treatment with CL097 (5 µg/ml) Xpress-TRIM21 was immunoprecipitated from cell lysates and association of TRIM21 with IRF5 isoforms was assessed by anti-Myc immunoblot. Normal mouse IgG (lanes 9 and 10) was used as negative control. WCL, whole cell lysate; H.C., Heavy Chain.

The effect of TRIM21 on IRF stability relies on its E3 ubiquitin ligase activity: by adding poly-ubiquitin chains on specific lysine residue(s) on the IRFs, TRIM21, like other E3 ubiquitin ligases, creates a signal that targets the activated transcription factor for proteasomal- or lysosomal-mediated degradation, thus achieving termination of signaling [16], [21], [30]. Having shown that all the isoforms interact with TRIM21 uniformly, we next assessed the ability of TRIM21 to ubiquitinate the single IRF5 isoforms. Myc-tagged IRF5 isoforms along with HA-ubiquitin were over-expressed in HEK-293T cells in presence or absence of Xpress-TRIM21. Following isolation of HA-ubiquitin-bound proteins from the cell lysates, the extent of ubiquitination for each isoform was determined by immunoblotting with anti-Myc antibody. As figure 3A shows, all IRF5 isoforms appear to be moderately ubiquitinated when co-transfected with ubiquitin alone (figure 3A, upper panel, lanes 3–6); however, ubiquitination dramatically increases in presence of TRIM21 for all isoforms, confirming that IRF5 is a substrate for TRIM21 ubiquitin-ligase activity (figure 3A, lanes 7–10). In order to investigate the mechanism by which ubiquitination may affect either IRF5 stability or activity, the extent of TRIM21-mediated IRF5 ubiquitination was assessed using ubiquitin mutants lacking lysines at position 48 or 63 and thus unable to form K48- or K63-linked poly-ubiquitin chains (figure S1), respectively, or mutants carrying only lysines at position 48 or 63 (figure S2). In all cases TRIM21 retained the ability to ubiquitinate IRF5 isoforms in presence of the various ubiquitin mutants, albeit with slightly different banding patterns, thus indicating that TRIM21 may target IRF5 with different types of ubiquitin chains and may thus have multiple roles in regulating IRF5 activity.

We next investigated whether the interaction between IRF5 and TRIM21 could be affected by TLR stimulation, previously shown to enhance TRIM21 affinity for its substrates, focusing in particular on the TLR7 pathway known to activate IRF5 and of primary importance in SLE [5], [16], [21], [31], [32]. THP-1 cells were stimulated with the TLR7 ligand Imiquimod and cell lysates were incubated with recombinant GST-PRY/SPRY TRIM21. Results shown in figure 3B (upper panels, lane 3) show that in the late phase of TLR7 stimulation the affinity of IRF5 for TRIM21 is slightly increased, suggesting therefore that TRIM21 can target IRF5 in this pathway. In keeping with the ability of Imiquimod to induce an interaction between TRIM21 and IRF5, TLR7 stimulation of PBMCs resulted in a time dependent degradation of IRF5 (figure 3B, lower panels, lanes 5 and 6), confirming that degradation can be induced following TLR-mediated activation.

In order to investigate how TLR7 stimulation would modulate the affinity of individual IRF5 isoforms for TRIM21, we overexpressed plasmids encoding TRIM21 and IRF5 isoforms in HEK-TLR7 cells. Following treatment with the TLR7 ligand CL097, preferred to Imiquimod given the enhanced ability of CL097 to activate IRF5 in this cell line (data not shown), TRIM21 was immunoprecipitated from cell lysates and association with IRF5 isoforms was assessed by anti-Myc immunoblot. As shown in figure 3C, TRIM21 interacted with each of the isoforms to a similar extent in resting cells (figure 3C, upper panel, lanes 1, 3, 5 and 7), with the interaction increasing in each case following TLR7 stimulation (figure 3C, upper panel, lanes 2, 4, 6 and 8).

### TRIM21 regulates IRF5 stability and activity in an isoform-specific manner

**Figure 4:**
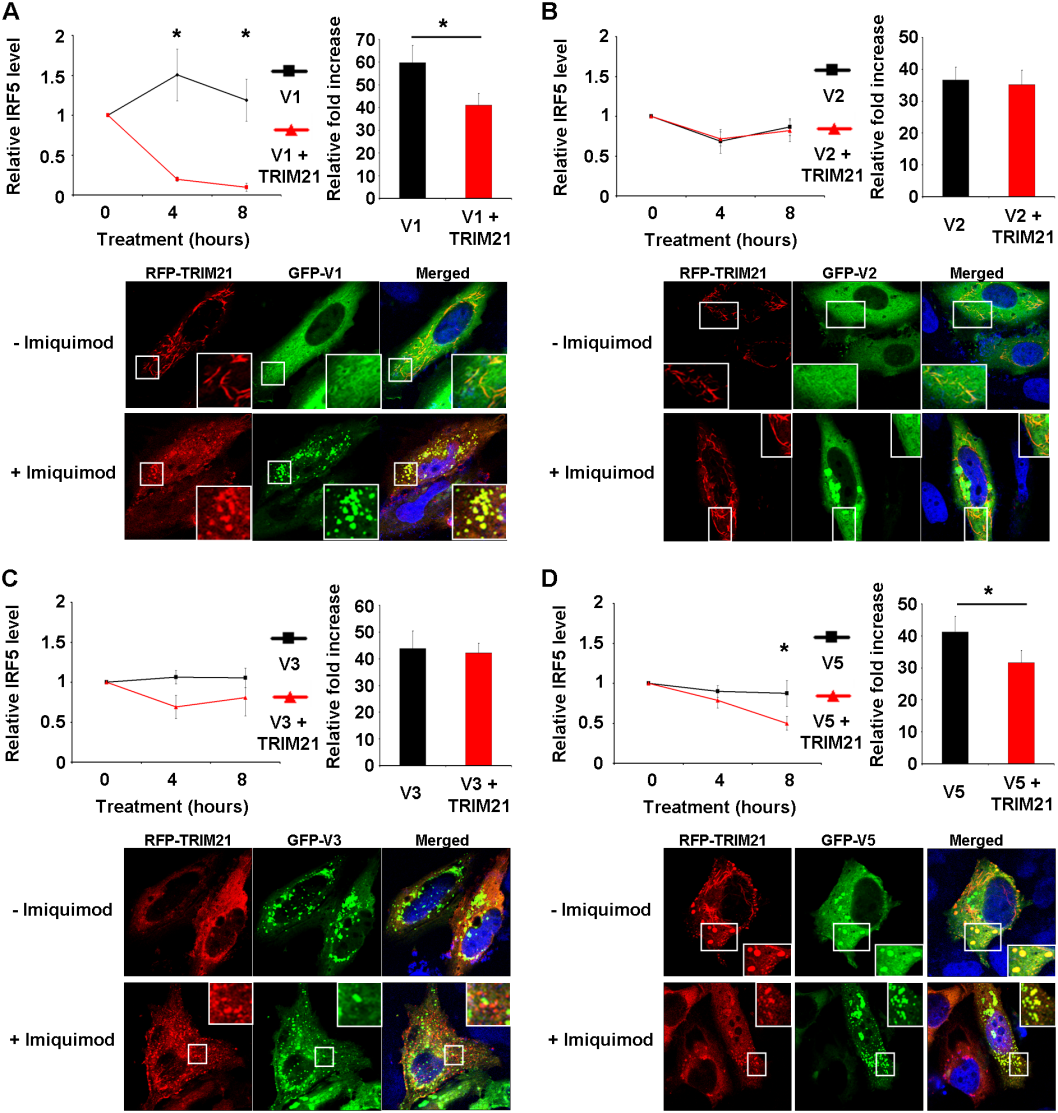
TRIM21 differentially regulates the stability of IRF5 isoforms. A–D, top left, HEK-TLR7 cells were transfected with Myc-tagged IRF5 isoforms (A, V1; B, V2; C, V3; D, V5) in presence or absence of Xpress-TRIM21. The day after transfection cells were treated with cycloheximide (100 µg/ml) in combination with CL097 (5 µg/ml) for the indicated times. Levels of IRF5, TRIM21 and α-Actinin were determined by immunoblot and levels of IRF5 normalized to α-Actinin were calculated and plotted, *p<0.05. A–D, top right, HEK293T were transfected with plasmids encoding the luciferase reporter gene under the control of the IFNA4 promoter, Myc-tagged IRF5 isoforms and MyD88 in presence or absence of Xpress-TRIM21. The TK-Renilla plasmid was used as internal control. Luciferase activity was measured 48 hours after transfection and normalized to renilla activity. Results are shown as fold activation over Empty Vector control, *p<0.05. A–D, bottom, HeLa cells were transfected with 1 µg of plasmids encoding GFP-tagged IRF5 (green) and mRFP-TRIM21 (red) and left untreated or stimulated with Imiquimod for 3 hours. Cells were fixed mounted in DAPI in order to visualize nuclei (blue) and images were taken under oil immersion at 63× magnification. Images shown are from a single experiment and are representative of three independent experiments.

Having shown that TLR7 stimulation promotes IRF5 degradation and interaction of IRF5 isoforms with TRIM21, we next investigated how TRIM21 affected the stability of the individual isoforms by performing a series of pulse-chase experiments in HEK-TLR7 cells. IRF5 isoforms were over-expressed in HEK-TLR7 cells in presence or absence of TRIM21 and, following treatment with the protein synthesis inhibitor cycloheximide, cells were stimulated with the TLR7 ligand CL097 and relative IRF5 protein levels were assessed by western blot (figure S3) and normalized to α-actinin levels. As figure 4A–D shows, TRIM21 overexpression in HEK-TLR7 cells treated with cycloheximide and CL097 promoted the degradation of IRF5-V1 at the early time point (figure 4A, top panel, left) and IRF5-V5 in the late phase of treatment (figure 4D, top panel, left), whilst no appreciable effect of TRIM21 on the stability of IRF5-V2 and IRF5-V3 was observed (figure 4B and C, top panels, left). Taken together these results therefore indicate that isoforms originating from alternative splicing (IRF5-V2 and IRF5-V3), lacking the first 48 nucleotides encoding the PEST domain, are resistant to TRIM21-mediated degradation following TLR7 stimulation, whilst the presence or absence of the 30 nucleotide insertion within the PEST domain encoding region has no effect on the stability of IRF5 isoforms. In keeping with the stability data, confocal analysis of GFP-IRF5 and RFP-TRIM21 subcellular localization in HeLa cells treated with Imiquimod reveals that IRF5 isoforms targeted for degradation (V1 and V5) co-localize with TRIM21 in vesicular structures which may represent sites of degradation of poly-ubiquitinated proteins such as autophagosomes/lysosomes (figure 4A and D, bottom panels), whilst no co-localization in such structures can be observed for the stable isoforms V2 and V3 (figure 4B and C, bottom panels).

TRIM21 was previously shown to inhibit IRF7- and IRF3-mediated activation of IFNα and IFNβ promoters [16], [21], and we thus next investigated whether the same regulatory mechanism could apply to IRF5 and whether differences could be observed between the different isoforms, given the differential ability of TRIM21 to selectively degrade only IRF5 isoforms originated by conventional splicing (IRF5-V1 and IRF5-V5). We therefore used reporter gene assays to measure IRF5 isoforms activity in presence or absence of TRIM21. HEK-293T cells were transfected with a reporter gene controlled by the IFNA4 promoter together with plasmids encoding IRF5 isoforms and MyD88 to mimic TLR-mediated IRF5 activation (figure 4A–D, upper panels, right). In keeping with the stability data, we observed significant TRIM21-mediated inhibition of IRF5-V1 and IRF5-V5 activity, whilst IRF5-V2 and IRF5-V3 activity was not affected by TRIM21 co-transfection. Taken together our results indicate that TRIM21 interacts with all isoforms of IRF5 thus far studied and that it contributes to TLR7-mediated destabilisation of IRF5 in an isoform-specific manner. Most importantly, the ability of TRIM21 to promote destabilisation of IRF5-V1 and -V5 translates to inhibitory effects on TLR7-mediated activation of the IFNA4 promoter. Thus TRIM21 can affect IRF5-mediated signal transduction and gene expression in an isoform specific manner.

## Discussion

Despite an increasing body of evidence suggesting that genetic variants in *IRF5* are linked to enhanced susceptibility to the autoimmune disease SLE, a comprehensive functional characterization of these variants is still missing. As one such polymorphism is a 30 nucleotide insertion in the PEST domain-encoding exon 6 of the *IRF5* gene, we investigated the stability of four IRF5 isoforms bearing different combinations of insertion/deletions in the PEST domain due to the presence or absence of the insertion and/or generated via alternative splicing. By investigating the molecular mechanism of IRF5 degradation following TLR stimulation we have identified IRF5 as a substrate of the E3 ubiquitin ligase TRIM21, previously shown to target other IRFs for degradation post-pathogen recognition [16], [20], [21]. Interestingly, analysis of the single isoforms revealed that IRF5 variants originating from alternative splicing (V2 and V3) and missing the first 48 nucleotides of the PEST domain-encoding region are resistant to TRIM21-mediated degradation and inhibition, thus suggesting that the enhanced expression of these isoforms in SLE patient monocytes may be as a result of decreased ability of TRIM21 to degrade them [23].

Previous studies have shown that TRIM21 interacts with its substrates via its C-terminal PRY/SPRY domain [16], [17], [19], [26], [27]. Whilst all TRIM proteins share a common structure composed of an N-terminal RING domain followed by one or two B-Box domains and a Coiled-Coil region, the C-terminal domain is more variable and considered to be important in mediating substrate specificity [33]. The PRY/SPRY domain in particular has evolved in parallel with adaptive immune mechanisms and is present in many TRIM members involved in regulation of the immune response (TRIM16, -20, -21, -22, -25, -27) or restriction of viral replication (TRIM5α) [34]–[36]. Several disease-associated mutations of *TRIM* genes have been identified in regions encoding this C-terminal domain, thus highlighting the importance of this protein region for substrate recognition and ultimately TRIM function [29]. As already observed for other IRF family members, our results demonstrate that the interaction between TRIM21 and IRF5 requires the PRY/SPRY domain, thus indicating a common mechanism for TRIM21 to target this family of transcription factors. Importantly, TLR-mediated phosphorylation of tyrosine residues in this domain was shown to be necessary to enhance affinity of TRIM21 for IRF3 [27]. The recombinant PRY/SPRY TRIM21 we used in this study, although an invaluable tool to examine the interaction properties of various overexpressed IRF5 isoforms or deletion mutants, likely does not mimic TLR-activated TRIM21. As such, the weakness of the interaction we observed between recombinant PRY/SPRY TRIM21 and endogenous IRF5 may suggest that TLR-induced post-translational modification of both IRF5 and TRIM21 is necessary to mediate a strong association between the two proteins.

When we analyzed the interaction properties of individual IRF5 isoforms we observed comparable levels of association between TRIM21 and all IRF5 isoforms investigated, suggesting that the IRF5 polymorphic region was not involved in mediating the interaction with TRIM21. Indeed, analysis of the interaction properties of various IRF5 deletion mutants indicated the C-terminal IAD domain (encoded by exons 7 to 9) to be necessary and sufficient to mediate the interaction with TRIM21, thus confirming that polymorphisms in the region encoded by *IRF5* exon 6 do not alter the affinity of IRF5 for TRIM21. Interestingly the interaction was enhanced following TLR7 stimulation for all isoforms suggesting, as mentioned previously, that TLR-mediated activation of IRF5 triggers post-translational modifications that increase IRF5 affinity for TRIM21. The C-terminal IAD domain in IRF5, which we show to mediate the interaction between IRF5 and TRIM21, has indeed been shown to undergo structural changes following TLR-mediated phosphorylation of conserved serine residues in this region. Phosphorylation-dependent dislocation of an autoinhibitory helix is necessary to expose IRF5 dimerization domain and to allow the formation of homo- and heterodimers which can then associate with other transcriptional co-activators such as CBP/p300 [12], [37]. Furthermore, analysis of the crystal structure of the closely related IRF3 and other IRF family members suggests the possibility that, in an inactive state, the N-terminal DNA binding domain of IRFs may be folded upon the C-terminal interaction domain [38]–[40]. Thus, virus-induced IRF phosphorylation could induce dislocation of C-terminal autoinhibitory structures and repositioning of the N-terminal DNA binding domain, resulting in unmasking of the DNA binding residues and the IAD interaction domain. In keeping with this hypothesis, we observed enhanced interaction with TRIM21 PRY/SPRY domain of IRF5 mutants lacking N-terminal domains (N1–N4) as compared to IRF5 full length, thus indicating an inhibitory effect of IRF5 N-terminal on IRF5-TRIM21 interaction. Interestingly, the phosphorylation-dependent switch from an autoinhibited form to the active one observed for IRF5 is shared by other members of the IRF family, such as IRF3 and IRF7, all of which are targeted by TRIM21 for degradation post-pathogen recognition [37], [39], [41]. Thus, regulatory mechanisms common to different IRFs suggest that phosphorylation, which allows for dimerization and activation of this family of transcription factors, also represents a signal for degradation, as already shown for IRF3, with TRIM21 emerging as the key E3 ubiquitin ligase targeting the IRF family [42]. It has recently been shown that an intact IAD is necessary to mediate IRF3 and IRF7 degradation by the rotavirus non-structural protein NSP1, suggesting that this conserved common region may be similarly targeted by cellular and viral E3 ligases in order to achieve termination of IRF-mediated signaling [43]. In this context, it is possible therefore that the IAD domain of IRF proteins, conserved in all members from IRF3 to IRF9, may be a common target for TRIM21-mediated degradation of this family of transcription factors.

With respect to a role for TRIM21 in regulating IRF5 stability, we observed enhanced IRF5 expression in cells depleted of TRIM21 by targeted shRNA silencing. Interestingly, expression of one of the genes controlled by IRF5, IL-6, was also enhanced in these samples, thus indicating that TRIM21 can regulate IRF5 turnover and consequently IRF5-mediated gene expression. In keeping with this, we observed that TRIM21 can dose-dependently inhibit IRF5-mediated activation of the IFNA4 promoter as analyzed by reporter gene assay. Collectively, our results therefore indicates that IRF5 is a novel target for TRIM21 and that dysregulation of TRIM21 activity in SLE may thus contribute to enhanced IRF5 levels and consequently to the enhanced levels of type I IFN and pro-inflammatory cytokines, in part regulated by IRF5, observed in SLE patients [44], [45]. Analysis of the single IRF5 isoforms examined revealed that, whilst all isoforms interacted with and were ubiquitinated by TRIM21 to a similar degree, their turnover rate presented differences, thus suggesting that ubiquitination may not be the sole determinant of IRF5 isoforms stability. In particular, we observed TRIM21-dependent degradation of IRF5 variants originating by conventional splicing (V1 and V5) following TLR7 stimulation, whilst IRF5 isoforms originating from alternative splicing (V2 and V3) were resistant to TRIM21-mediated degradation. Confocal analysis of IRF5 and TRIM21 subcellular localization in TLR7-stimulated cells provided a useful insight into the probable mechanism of TRIM21-mediated degradation of IRF5 and potentially explains the differences observed between the various IRF5 isoforms. We observed in fact co-localization of the conventionally spliced and unstable isoforms V1 and V5 with TRIM21 in vesicular cytoplasmic structures resembling autophagosomes/lysosomes, previously shown to mediate degradation of intracellular ubiquitinated proteins [46], [47], whilst no co-localization of TRIM21 with the stable isoforms V2 and V3 in such structures was observed. The ubiquitin-binding protein p62 was previously shown to be necessary for formation and degradation of polyubiquitin-containing bodies by autophagy [48], and interestingly, p62 cooperates with TRIM21 in orchestrating IRF8 degradation. Thus, while TRIM21-mediated ubiquitination of IRF8 was shown to initially enhance its activity, p62 binding to ubiquitinated IRF8 in the late phase of the response was shown to be necessary to promote its degradation [17], [20]. Our results suggest that a similar mechanism may be in place in regulating IRF5 stability, and the differences observed between the various IRF5 isoforms may therefore reflect differences in their affinity for p62, since we did not observe differences in the affinity of IRF5 isoforms for TRIM21. Further studies in the role of p62 in regulating the stability of IRF5 isoforms will help to precisely define the mechanism of IRF5 degradation.

Regardless of the specific mechanism involved, the finding that alternatively spliced isoforms have increased stability in TLR7-stimulated cells is of particular relevance in the context of SLE, since elevated levels and activity of spliceosome components have been observed in PBMCs from SLE patients indicating therefore that the more stable alternatively spliced IRF5 isoforms (IRF5-V2 and -V3) may be over-represented in SLE patients’ immune cells [12]. Indeed, Stone *et al* recently reported that the stable isoform IRF5-V2 mRNA is significantly overexpressed in monocytes from SLE patients as compared to controls [23]. Furthermore, the same study identified a large number of novel IRF5 variants, many of which, like the stable isoforms V2 and V3 investigated here, are generated by alternative splicing of the 5′ region of exon 6 and are therefore likely to escape TRIM21-mediated negative regulation possibly due to alterations in their PEST domain structure. In keeping with the stability data, analysis of the effect of TRIM21 on the ability of IRF5 isoforms to activate the IFNA4 promoter indicated that the activity of IRF5 isoforms V1 and V5, targeted by TRIM21 for degradation in TLR7-activated cells, is inhibited in presence of TRIM21, whilst the stable isoforms V2 and V3 are resistant to TRIM21-mediated degradation and can not be inhibited by TRIM21.

Taken together, our results indicate that interaction of IRF5 with TRIM21 and its subsequent ubiquitination occurs regardless of the isoforms examined here. However, the effects of TRIM21 are indeed isoform specific, with V1 and V5 being destabilised by TRIM21 whilst V2 and V3, which arise from alternative splicing of exon 6 and have therefore altered PEST domain structure, are stable in presence of TRIM21. Our finding that alternative splicing of the IRF5 transcript results in expression of isoforms, like V2 and V3 examined in this study, able to escape TRIM21-mediated degradation and therefore not inhibited by TRIM21 upon TLR activation, suggests that the presence of SLE-specific, degradation-resistant IRF5 isoforms may mediate the enhanced production of type I IFN and proinflammatory cytokines known to play a critical role in SLE development and pathogenesis.

## Supporting Information

Figure S1 **TRIM21 ubiquitinates IRF5 isoforms with both K48- and K63-linked polyubiquitin chains.** Myc-tagged IRF5 isoforms and HA-Ubiquitin wild type, K48R or K63R mutants were overexpressed in HEK-293T in presence or absence of Xpress-TRIM21. Lysates were incubated with HA agarose and the extent of IRF5 ubiquitination was assessed by anti-Myc immunoblot (top panels). Expression of IRF5 and TRIM21 in the Whole Cell Lysate (WCL) is shown in the bottom panels. A, IRF5-V1 (IRF5 lysates membrane was reblotted with anti-Xpress and *indicates the residual Myc signal detected in the Xpress immunoblot); B, IRF5-V2; C, IRF5-V3; D, IRF5-V5.
(TIF)

Figure S2 **TRIM21 ubiquitinates IRF5 isoforms with both K48- and K63-linked polyubiquitin chains.** Myc-tagged IRF5 isoforms and HA-Ubiquitin R48K (panel A) or R63K (panel B) mutants were overexpressed in HEK-293T in presence (lanes 7–10) or absence (lanes 3–6) of Xpress-TRIM21. Lysates were incubated with HA agarose and the extent of IRF5 ubiquitination was assessed by anti-Myc immunoblot (top panels). Expression of TRIM21 in the whole cell lysates (WCL) is shown on the bottom panels. *indicates the residual Myc signal detected in the Xpress immunoblot.
(TIF)

Figure S3 **TRIM21 differentially regulates the stability of IRF5 isoforms - Western blot analysis.** A–D, HEK-TLR7 cells were transfected with Myc-tagged IRF5 isoforms (A, V1; B, V2; C, V3; D, V5) in presence or absence of Xpress-TRIM21. The day after transfection cells were treated with cycloheximide (100 µg/ml) in combination with CL097 (5 µg/ml) for the indicated times. Levels of IRF5, TRIM21 and α-Actinin were determined by immunoblot.
(TIF)
