# Supplementary figures and images for: TRIpartite Motif 21 (TRIM21) Differentially Regulates the Stability of Interferon Regulatory Factor 5 (IRF5) Isoforms
Source: PLoS One. 2014 Aug 1;9(8):e103609. doi: 10.1371/journal.pone.0103609 (PMC4118895; doi:10.1371/journal.pone.0103609)

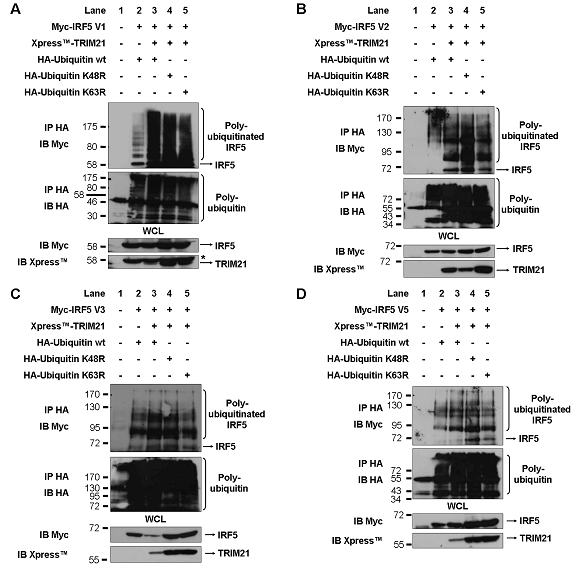

Supplement: Figure S1 — TRIM21 ubiquitinates IRF5 isoforms with both K48- and K63-linked polyubiquitin chains. Myc-tagged IRF5 isoforms and HA-Ubiquitin wild type, K48R or K63R mutants were overexpressed in HEK-293T in presence or absence of Xpress-TRIM21. Lysates were incubated with HA agarose and the extent of IRF5 ubiquitination was assessed by anti-Myc immunoblot (top panels). Expression of IRF5 and TRIM21 in the Whole Cell Lysate (WCL) is shown in the bottom panels. A, IRF5-V1 (IRF5 lysates membrane was reblotted with anti-Xpress and *indicates the residual Myc signal detected in the Xpress immunoblot); B, IRF5-V2; C, IRF5-V3; D, IRF5-V5. (TIF) [file pone.0103609.s001.tif]

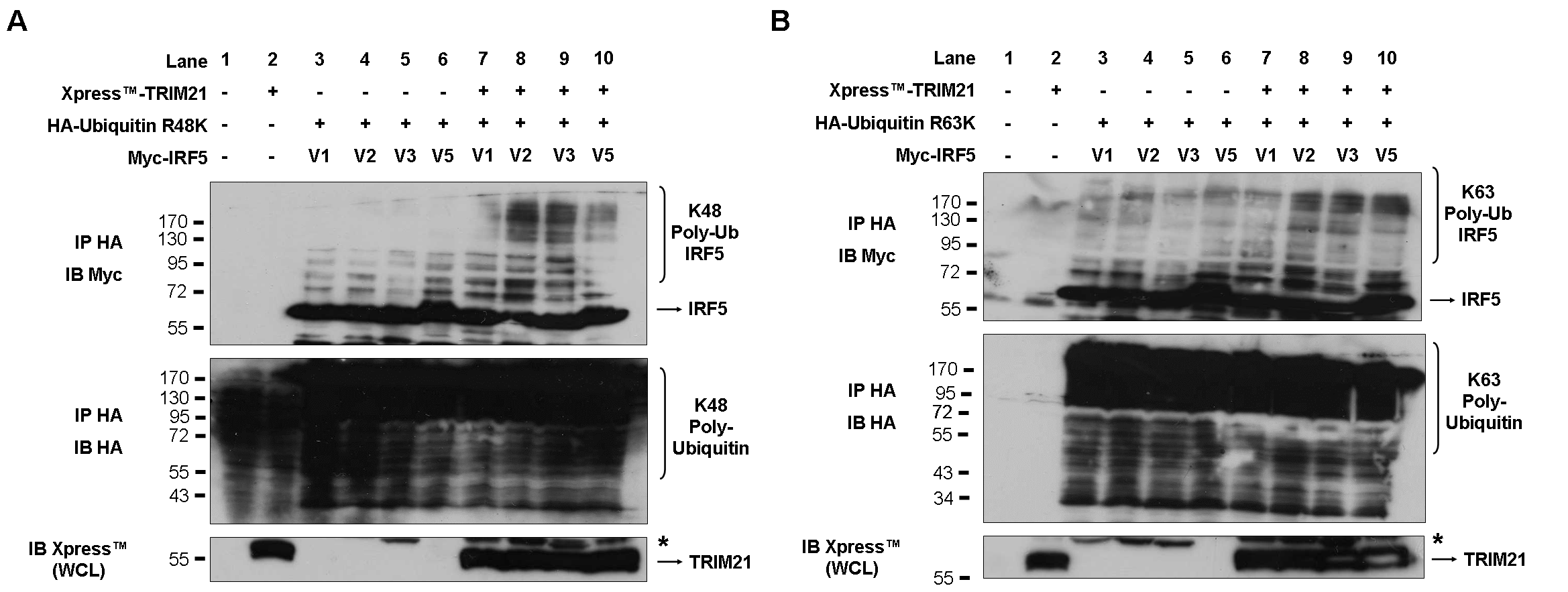

Supplement: Figure S2 — TRIM21 ubiquitinates IRF5 isoforms with both K48- and K63-linked polyubiquitin chains. Myc-tagged IRF5 isoforms and HA-Ubiquitin R48K (panel A) or R63K (panel B) mutants were overexpressed in HEK-293T in presence (lanes 7–10) or absence (lanes 3–6) of Xpress-TRIM21. Lysates were incubated with HA agarose and the extent of IRF5 ubiquitination was assessed by anti-Myc immunoblot (top panels). Expression of TRIM21 in the whole cell lysates (WCL) is shown on the bottom panels. *indicates the residual Myc signal detected in the Xpress immunoblot. (TIF) [file pone.0103609.s002.tif]

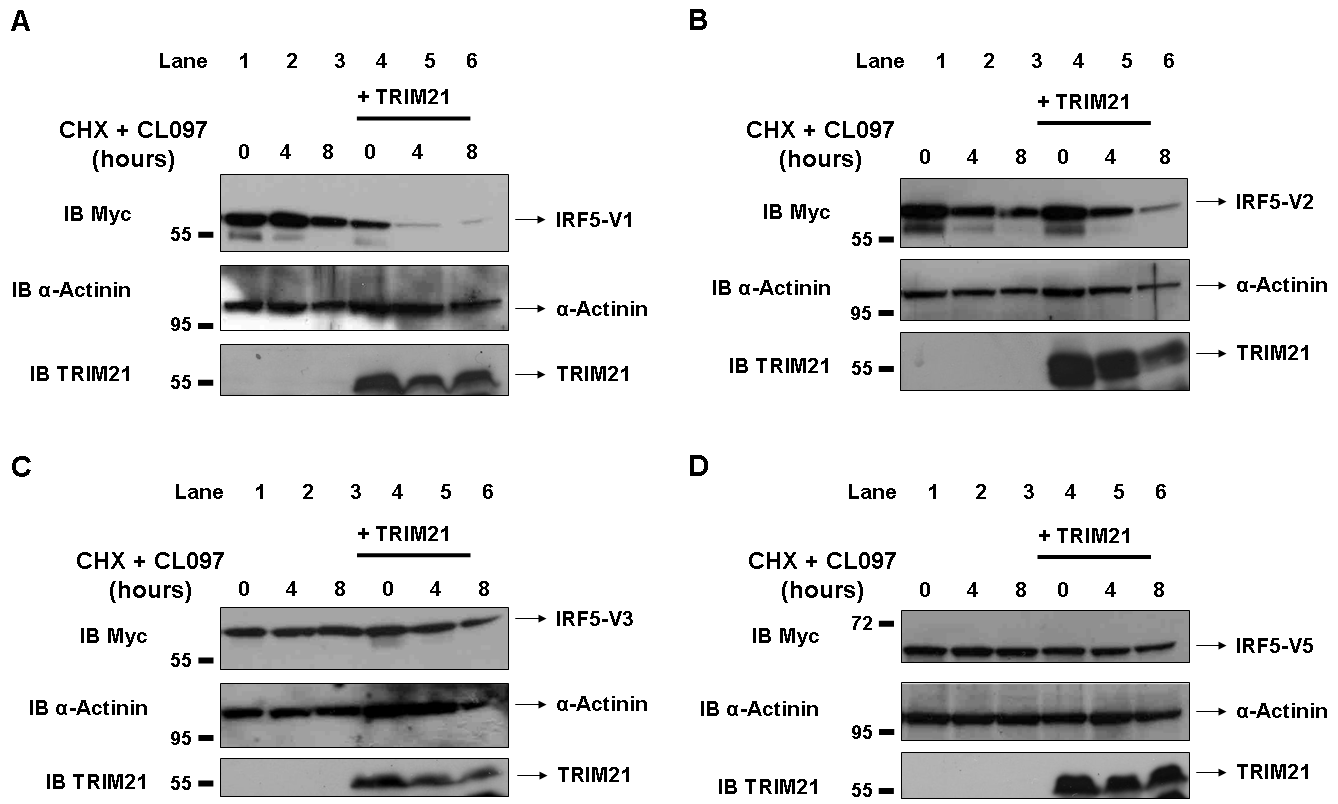

Supplement: Figure S3 — TRIM21 differentially regulates the stability of IRF5 isoforms - Western blot analysis. A–D, HEK-TLR7 cells were transfected with Myc-tagged IRF5 isoforms (A, V1; B, V2; C, V3; D, V5) in presence or absence of Xpress-TRIM21. The day after transfection cells were treated with cycloheximide (100 µg/ml) in combination with CL097 (5 µg/ml) for the indicated times. Levels of IRF5, TRIM21 and α-Actinin were determined by immunoblot. (TIF) [file pone.0103609.s003.tif]
